# Effects of Glutathione S-Transferase Gene Polymorphisms and Antioxidant Capacity per Unit Albumin on the Pathogenesis of Chronic Obstructive Pulmonary Disease

**DOI:** 10.1155/2017/6232397

**Published:** 2017-08-30

**Authors:** Tinghui Cao, Naijin Xu, Zhen Wang, Hui Liu

**Affiliations:** ^1^College of Medical Laboratory, Dalian Medical University, Dalian 116044, China; ^2^Department of Chemistry, Dalian Medical University, Dalian 116044, China; ^3^Department of Clinical Laboratory, First Affiliated Hospital of Dalian Medical University, Dalian 116011, China

## Abstract

**Objectives:**

To study the effects of *GSTM*1, *GSTT*1 gene polymorphisms, and organism antioxidant capacity and related indicators such as antioxidant capacity per unit of albumin (AC/ALB) on chronic obstructive pulmonary disease (COPD).

**Methods:**

Using polymerase chain reaction technology, *GSTM*1 and *GSTT*1 gene polymorphisms were detected in 33 COPD patients and 33 healthy people. The total antioxidant capacity (TAC) found in serum was determined using the I_2_/KI potentiometric, KMnO_4_ microtitration, and H_2_O_2_ potentiometric methods. The AC/ALB was defined as the TAC divided by the serum albumin concentration. Logistic regression analysis was carried out with biochemical screening indices, which was found to be closely related with the incidence of COPD.

**Results:**

The *GSTM*1 and *GSTT*1 gene deletion rate in the COPD group was significantly higher than that in the control group (*P* < 0.05). The differences in serum TAC between the COPD and control groups, *GSTM*1 (+) and *GSTM*1 (−) groups, and *GSTT*1 (+) and *GSTT*1 (−) groups were statistically significant (*P* < 0.001). In addition, there was a significant difference in the AC/ALB between the COPD and control groups (*P* < 0.05). Logistic regression analysis showed that the incidence of COPD was closely related to the AC/ALB (*P* < 0.05).

**Conclusions:**

*GSTM*1 and *GSTT*1 gene polymorphisms are closely correlated with the pathogenesis of COPD, while the AC/ALB plays a decisive role in the occurrence and development of COPD.

## 1. Introduction

Chronic obstructive pulmonary disease (COPD) is characterized by decreased expiratory flow, increased airway resistance, and pulmonary hyperinflation. The airflow limitation associated with COPD is not fully reversible and is progressive. In recent years, due to the gradual increase in the COPD prevalence and mortality due to the disease, it has become an important public health problem [1]. Expected to become the world's third most fatal disease by 2030, there are currently 6 million COPD patients globally [2]. Therefore, in-depth research into the pathogenesis of COPD is of great significance for the prevention and treatment of the disease.

**Figure 1:**
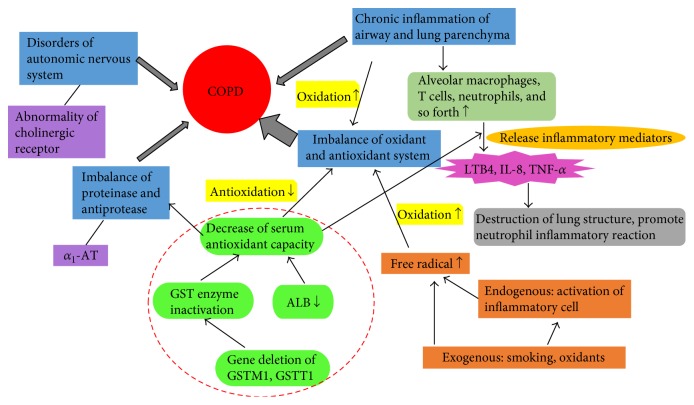
Pathogenesis of chronic obstructive pulmonary disease (COPD).

The etiology of COPD is complex. The prevailing view is that airway remodeling is the main cause of irreversible airflow limitation and progressive disease development. Airway remodeling is associated with the process of airway inflammation, which predominately occurs in the lungs [3]. Since the pathogenesis of COPD is not yet fully understood [4], such research is typically focused on four aspects which include (1) lung inflammation, (2) the imbalance of lung protease and anti-proteases, (3) oxidation and antioxidation imbalances, and (4) autonomic nervous system dysfunction [5], such as abnormal cholinergic receptor distribution (Figure 1).

It is generally believed that COPD is characterized by chronic inflammation of the airway, lung parenchyma, and pulmonary blood vessels. T lymphocytes and neutrophils, and in some patients, eosinophilia increased in different parts of the lung containing alveolar macrophages [6]. The activated inflammatory cells, through the release of various inflammatory mediators such as leukotriene B4, interleukin 8, and tumor necrosis factor *α* [7], either destroy the lung structure or promote a neutrophil inflammatory reaction. Currently, *α*_1_-antitrypsin (*α*_1_-AT) is the only confirmed antiprotease associated with COPD [8]. If *α*_1_-AT gene-deficient patients smoke, they have higher COPD attack rates and also present with COPD at an earlier age. Oxidative stress is the *in vivo* imbalance between oxidation and antioxidation. When induced, antioxidant capacity decreases, which leads to neutrophil infiltration and increased protease secretion. This also results in a large number of intermediate products, such as reactive oxygen species (ROS). The body simultaneously secretes antioxidants to eliminate harmful oxidative substances. The imbalance between oxidants and antioxidants leads to important molecular and cellular damage, eventually affecting the whole body [9]. Because the lungs are exposed to the air through the bronchi, when coupled with high oxidative stress, a large number of ROS-free radicals are released [10, 11]. ROS can directly attack the polyunsaturated and unsaturated fatty acids in lung cells, which results in membrane lipid peroxidation. Oxidants can also directly damage the matrix components of the lung, such as elastin and collagen, and also inhibits the synthesis and repair of elastin. This results in the increased proteolysis of matrix components and the rate at which emphysema develops.

Some studies have shown that genetic predisposition and oxidative stress play a key role in triggering lung cell destruction or dysfunction [12, 13]. For oxidative stress, the normal human body has an antioxidant mechanism that includes the microsomal peroxidation enzyme, heme oxygenase, glutathione S-transferase (GST), cytochrome P450 enzyme family, and extracellular superoxide dismutase [14]. These enzymatic antioxidants catalyze a variety of chemical substances, their metabolites, and toxic substances produced by oxidative stress, in order to prevent the destruction of cells due to ROS. However, genetic variation can decrease the presence of antioxidants. This destroys the dynamic balance between oxidation and antioxidation, leading to oxidative damage.

GST is a phase II biotransformation enzyme and is divided into cytoplasmic, membrane, mitochondrial, and leukotriene C4 synthases. Cytoplasmic GSTs include, among others, *α*, *μ*, *π*, *σ*, *θ*, *κ*, ζ, and Ω subtypes [15]. The toxic electrophilic substances produced by oxidative stress are eliminated by the conjugating reaction of reduced glutathione and GSTs [16]. They play an important role in the response of organisms to environmental physical and chemical stimulation and protect the cell, protein, and nucleic acid from free radical damage. Recent studies [17] have found that GST enzymes bind to a variety of toxic substances in cigarettes (such as oxidizing agents/free radicals) and act as substrates for biotransformation metabolism thereby protecting cells from the damage caused by cytotoxic and carcinogenic factors. GSTs are thus important for the protection of airway and alveolar epithelial cells and play an important role in the prevention and treatment of COPD. The catalytic function of GSTs, which is closely related to the susceptibility to COPD, is influenced by the polymorphic nature of several *GST* genes. At present, research is commonly focused on the *GSTM*1 and *GSTT*1 genes. *GSTM*1 can detoxify benzo pyrene diol epoxide, and *GSTT*1 can remove the conjugated lipid peroxide and halogenated compounds. Previous studies [18, 19] have suggested that *GSTM*1 and *GSTT*1 gene deletions may be related to the occurrence and development of COPD. *GSTM*1 and *GSTT*1 are, respectively, located in chromosome 1p13.3 and 22q11.23, both existing as null alleles. GST enzyme activity is lost or reduced in individuals with deletions in the *GSTM*1 or *GSTT*1 genes, leading to an increased susceptibility to environmental factors causative of COPD. *GSTM*1 or *GSTT*1 gene deletions are additionally associated with diseases such as bronchial asthma [20], rheumatoid arthritis [21], systemic sclerosis [22], Parkinson's disease [23], diabetes mellitus [24], and several forms of cancer [25–28].

In this study, the serum total antioxidant capacity (TAC) was measured by three methods: the I_2_/KI potential method, KMnO_4_ microtitration method, and H_2_O_2_ potential method. The results of these analyses indicate that oxidative stress is an important etiological factor of COPD. Detection of an organism's oxidative stress, as described in previous studies [29–31], was mainly through the concentration determination of glutathione, superoxide dismutase, free radicals, and other substances and was focused on the antioxidant capacity at a cellular level. However, determination of serum TAC via combined detection methods, as presented here, measures antioxidative capacity at a macro level. In this study, *GSTM*1 and *GSTT*1 gene polymorphisms and resultant TAC of COPD patients were measured. By catalyzing the binding of GSH and various oxidants, GST enzymes promote the antioxidant activity of GSH. GST gene expression may thus be associated with biochemical pathways through GSH activity. Glucose [32] and albumin [33] have been shown to influence antioxidation activity in serum, and total serum protein was shown to include both albumin and globin. If the antioxidant properties of proteins or sugars can be affected by the presence of certain oxidants and antioxidants, then the changes may contribute to the development of diseases. Biochemical indicators in serum are likely to be biomarkers of oxidative status [34]. In this study, the associated effects of biochemical indices on antioxidant capacity were determined, and the related antioxidant capacity index was obtained. Finally, the antioxidant capacity index and the incidence of COPD underwent correlative analysis in order to explore the relationships between the gene polymorphism and disease outcome.

## 2. Materials and Methods

### 2.1. Clinical Specimen Collection

A series of 33 randomly selected COPD patients (26 men and seven women with a mean age of 73.42 ± 13.76 years) and 33 healthy subjects (26 men and seven women with a mean age of 69.76 ± 13.00 years) were recruited at the First Affiliated Hospital of Dalian Medical University. Neither the COPD patients nor the controls had long smoking histories. COPD was diagnosed following Global Initiative for Chronic Obstructive Lung Disease criteria [35], based on clinical signs and symptoms, laboratory tests, and imaging studies. Patients with symptoms of chronic progressive dyspnea, cough, and sputum received pulmonary function testing. A postbronchodilator FEV1/FVC ratio < 70% confirmed the presence of persistent airflow limitation and confirmed the diagnosis of COPD. Patients with oxygen inhalation and corticosteroid treatment within the previous month, or with serious comorbidities including diabetes mellitus, cardiovascular or cerebrovascular diseases, chronic liver or kidney disease, or tumors were excluded. The clinical characteristics and spirometric parameters of the study subjects are shown in Table 1. The control and study groups were matched by age and sex and recruited on the same day. The experimental specimens included serum and anticoagulated blood samples, which were collected, packaged, and stored at −20°C on the same day. Samples were thawed at room temperature before being analyzed. The related biochemical examination indices of all COPD patients and healthy controls were recorded while collecting specimens. The study was approved by the Ethics Committee of Dalian Medical University.

## 3. Methods

### 3.1. Detection of *GSTM*1 and *GSTT*1 Gene Polymorphisms

The whole blood DNA Extraction Kit (E.Z.N.A.TM blood DNA Kit, Omega Bio-Tek Inc., Norcross, GA, USA) was used to extract genomic DNA from anticoagulated whole blood collected from COPD patients and healthy controls. Extracted genomic DNA was stored at −20°C. The genomic DNA was amplified by PCR technology using *GSTM*1 gene primers (Forward, 5′-GAACTCCCTGAAAAGCTAAAGC-3′; Reverse, 5′-GTTGGGCTCAAATATACGGTGG-3′), *GSTT*1 gene primers (Forward, 5′-TTCCTTACTGGTCCTCACATCTC-3′; Reverse, 5′-TCACCGGATCATGGCCAGCA-3′), and reference gene *β*-actin primers (Forward, 5′-AATGTGAACATGTGGGACTTTGTG-3′; Reverse, 5′-CGCCAGTTCAGGACATTAGGAC-3′). Using the aforementioned primer pairs, the PCR products resulted in 215, 480, and 92 bp gene fragments, respectively. Using a 25 *μ*L reaction system (1 *μ*L of gene template, 0.5 *μ*L of *GST* gene forward and reverse primers, 0.5 *μ*L of reference gene forward and reverse primers, and 12.5 *μ*L of 2 × Power Taq PCR Master Mix, 9.5 *μ*L of ddH_2_O), the reaction conditions included initial denaturation (95°C, 5 min), denaturation (94°C, 30 s), annealing (63°C, 30 s), and extension (72°C, 30 s). A total of 35 cycles were performed. *GSTM*1 and *GSTT*1 gene regions were, respectively, amplified for each DNA specimen.

Following the PCRs, a 1.5% agarose gel, incorporating Genecolour II nucleic acid dye, was prepared. Gene expression results for each specimen were observed under a UVPC-80 gel imaging system (UCP Inc., San Jose, CA, USA).

### 3.2. Detection of Serum TAC

#### 3.2.1. Determination of Serum TAC via the I_2_/KI Potential Method

The I_2_/KI solution (0.1 mol/L of I_2_ and 0.4 mol/L of KI) was dispensed with a concentration ratio of 1 : 4. Samples were mixed in the following order: 100 *μ*L of tested serum, 20 *μ*L of I_2_/KI electric liquid, and 2880 *μ*L of deionized water. Mixed samples were placed at room temperature in the dark for 1 h. The oxidation-reduction potential (ORP) values at 120 s were then determined [36].

#### 3.2.2. Determination of Serum TAC via the KMnO_4_ Microtitration Method

In total, 80 *μ*L of a 0.005 mol/L KMnO_4_ solution and 20 *μ*L of a 10-fold dilution of tested serum were added to each well of a 96-well ELISA plate. Plates were turbulent mixed and then held in a water bath at 37°C in the dark for 30 min. Absorbance was measured at OD_570_ using a microplate reader [37].

#### 3.2.3. Determination of Serum TAC via the H_2_O_2_ Potential Method

A volume of 20 *μ*L per test serum was mixed with a 30% H_2_O_2_ solution to a total volume of 3000 *μ*L. Following storage at room temperature in the dark for 30 min, 120 s ORP values were obtained.

Antioxidant capacity measurements were determined from serum samples from both COPD patients and healthy controls using each of the aforementioned three methods. The results of the three methods were, respectively, converted into *Z* values as indicated in the following formula:

(1) $$\begin{array}{c} Z=\frac{test\;value‐mean}{S} \end{array}$$

(note: mean and standard deviation (*S*) were obtained from healthy control data).

The median *Z* value was obtained through the following calculation:

(2) $$\begin{array}{c} Z\left(M\right)=median\left[Z\frac{I_{2}}{KI},Z\left({KMnO}_{4}\right),Z\left(H_{2}O_{2}\right)\right]. \end{array}$$

The *Z* (*M*) value represents the TAC of the tested serum.

### 3.3. Correlation between Serum Biochemical Parameters and TAC

This study investigated serum markers possibly related to TAC, including glucose (GLU), albumin (ALB), and total protein (TP). ALB, GLU, and TP concentrations were determined with an autoanalyzer (Hitachi 7600-110, Tokyo, Japan) using standard commercial reagent kits. ALB (g/L) was assayed using the bromocresol green method, GLU (mmol/L) was assayed using the hexokinase method, and TP (g/L) was assayed using the biuret method.

Differences in marker concentrations and the antioxidant capacity of each marker related index, that is, the antioxidant capacity per unit albumin (AC/ALB) in COPD patients and control groups, were compared. Antioxidant status was described by the associations of antioxidant concentration, *GST* gene polymorphisms, and TAC.

### 3.4. Statistical Analysis

The experimental data were analyzed by SPSS version 13.0 statistical analysis software, and an *α* = 0.05 was used as the test level. Differences in *GSTM*1 and *GSTT*1 gene polymorphisms between the COPD patients and the healthy control group, as observed through electrophoretic imaging, were determined by *χ*^2^ testing. Independent sample *t*-tests were used to compare the *Z* (I_2_/KI), *Z* (H_2_O_2_), *Z* (KMnO_4_), and *Z* (M) values, as well as the differences in serum TAC (*Z* (M)) between COPD and healthy individuals. An independent sample *t*-test was also used to determine the differences in serum TAC associated with different *GST* genotypes. By comparing each index in the COPD patients and the control group, relevant indicators showing significant statistical difference could be selected. The relationship between the morbidity condition, antioxidant capacity per unit-related index, and *GST* gene polymorphisms was analyzed by logistic regression.

## 4. Results

### 4.1. The Relationship between *GSTM*1 and *GSTT*1 Gene Polymorphisms and COPD

**Figure 2:**
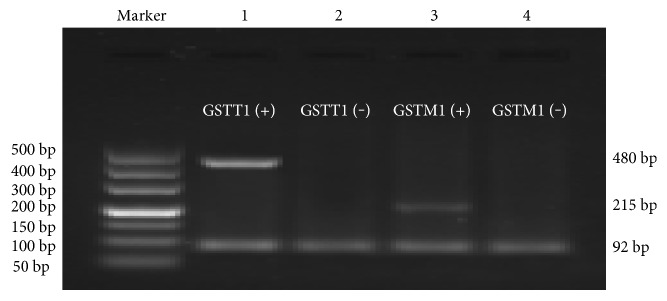
PCR amplification results of *GSTM*1 and *GSTT*1 genes.

PCR results of all specimens, as visualized through agarose gel electrophoresis, expressed a 92 bp reference gene (*β*-actin) band. These results showed normal amplification reactions. However, polymorphic expression of *GSTM*1 and *GSTT*1 genes were observed. *GSTM*1 (+) PCR amplification results showed a band of 215 bp, while *GSTM*1 (−) specimens lacked this band. Similarly, PCR amplification of *GSTT*1 (+) specimens showed a band of 480 bp, while *GSTT*1 (−) specimens lacked the 480 bp band. In Figure 2, the first and second lanes and the third and fourth lanes, respectively, were the amplification results of *GSTT*1 and *GSTM*1 genes. Representing the *GSTT*1 (+) genotype, the first lane indicated the presence of the 480 bp band, while the second lane, representing the *GSTT*1 (−) genotype, indicated amplification of the reference gene only. A 215 bp band representing the *GSTM*1 (+) genotype was observed in lane three, while the fourth lane, showing the presence of only the reference gene band, represents the *GSTM*1 (−) genotype. The differences between *GSTM*1 and *GSTT*1 gene polymorphisms across the COPD and control groups were statistically significant (*P* < 0.05) (Table 2).

### 4.2. Analysis of Serum to TAC with COPD Disease and *GST* Gene Polymorphisms

The I_2_/KI potential, KMnO_4_ microtitration, and H_2_O_2_ potential methods were used to conjunctively detect the TAC of serum (*Z* (M)). After statistical analysis, the TAC of tested specimens was significantly different between groups (*P* < 0.001) (Table 3). In addition, the TAC of *GSTM*1 or *GSTT*1 gene deletion subjects was significantly lower than that of the positive gene subjects (the higher the *Z* value, the lower the antioxidant capacity) (Table 4).

### 4.3. Correlation of Related Indices and TAC

Comparing related indicators between the COPD and control groups (Table 5), it was found that albumin (ALB) concentrations were significantly different (*P* < 0.001). Determined by the significant differences in TAC between groups (*P* < 0.001) (Table 3), a new index “AC/ALB,” that is, AC/ALB = *Z* (M)/ALB, was constructed taking into consideration the antioxidant capacity of per unit of albumin. Analyzing this ratio between the COPD and control groups (Table 6), the antioxidant capacity per unit of albumin between the two groups was found to be significantly different (*P* < 0.001).

Logistic regression was used to analyze the relationship between COPD and *GSTM*1, *GSTT*1, and the *Z* (M)/ALB. It was concluded that COPD was closely related to *Z* (M)/ALB. The tested model was statistically signficant. (*P* < 0.05). Parameter estimations and test results are shown in Table 7. From this, it can be seen that the *Z* (M)/ALB indicator was statistically significant and that a larger proportion of people were more susceptible to COPD. Due to the higher *Z* (M) value and lower antioxidant capacity, the decrease in the AC/ALB is a risk factor for COPD.

## 5. Discussion

This experimental study adopted a case-control research method considering clinically evaluated COPD patients and normal control subjects as research participants. In order to determine the relationship between COPD, gene polymorphism, and antioxidant capacity, the differences in frequency between these factors were evaluated in case and control groups.

Human cytoplasmic *GST* expresses genetic polymorphisms, such as those caused by deletions in the *GSTM*1 or *GSTT*1 genes, which results in protein deficiencies, a change in the activity of GST antioxidant enzymes and an increased susceptibility to oxidative stress. Gene polymorphisms associated with *GSTM*1 and *GSTT*1 were divided into wild-type and deletion-type polymorphisms (Figure 2). The results indicated that the *GSTM*1 (−) and *GSTT*1 (−) genotypes were observed at a higher frequency in the COPD group when compared to the control group (*P* < 0.05) (Table 2). These results suggest that the deletion of the *GSTM*1 or *GSTT*1 gene may be a risk factor for COPD. This result is consistent with related research [38, 39].

At the same time, the serum antioxidant capacity of COPD patients and the control group was determined through the combination of three methods. Since the median is unaffected by extreme values, unlike the mean, the *Z* (M) value was used to evaluate the TAC of the body (Table 3). The repeatability, linearity, and relative resolution of the I_2_/KI potential, KMnO_4_ microtitration, and H_2_O_2_ potential methods is good. Additionally, since iodine, KMnO_4_, and H_2_O_2_ are strong oxidizing substances, each can react with antioxidants in human serum to reflect serum antioxidant capacity. At present, no single method can measure the normal range of total serum antioxidant capacity in a healthy body and thereby necessitates the use of multiple, verified methods. Therefore, in this study, three kinds of methods were jointly used to evaluate redox states. In analyzing the relationship between serum TAC with COPD and *GST* gene polymorphisms, it was found that the *Z* (M) values in the COPD group were significantly higher than those of the control group (*P* < 0.001) (Table 3). Due to the lower antioxidant capacity associated with a higher *Z* (M) value, serum TAC in COPD patients was lower than that of healthy people. Furthermore, *Z* (M) values in subjects with deletions in either the *GSTM*1 or *GSTT*1 genes were higher than those in the wild-type group (Table 4). This showed that the TAC in the *GST* gene deletion group is lower than that in the wild-type group. Therefore, *GSTM*1 and *GSTT*1 genes have a protective function. Normal expression of these genes can reduce the body's injury from oxidative stress and decrease the risk of COPD. Identifying individuals with known COPD-associated *GST* polymorphisms may therefore be of great significance for the prevention, diagnosis, and treatment of COPD.

The classic antioxidant system can be divided into two categories [40]. One of these categories includes genes encoding macromolecular antioxidant enzymes such as catalase and glutathione antioxidant enzymes. These enzymes can remove ROS, thereby protecting cells from free radical attack. The second category includes small organic molecules such as vitamins C and E. Under physiologically normal conditions, the formation and removal of free radicals in the body is in a dynamic balance. Both too many and too few free radicals can have adverse effects on the body. Sources of free radicals mainly include exogenous factors, such as stimuli and pollutants in the environment (cigarette smoking, ozone, etc.), and endogenous factors such as inflammatory cell activation. When an oxidative stress injury occurs, a large number of reactive oxygen-free radicals and lipid peroxides are created. This increases the production of ROS in cells and decreases the ability of scavenging. Subsequently, the antioxidant capacity is decreased, thereby resulting in the damage of cells. For example, in the smoking process, oxidants result in the accumulation of actin-producing neutrophils. This changes the neutrophil cytoskeleton which increases the free neutrophils present in the pulmonary circulation system. This induces the release of ROS [41]. Many studies have shown that oxidative stress and free radicals may be associated with an increased susceptibility to a variety of diseases [42–45]. In the pathogenic process of COPD, gene regulation mediated by oxidants can enhance the expression of inflammatory genes and decrease the level of glutathione (GSH), thus promoting the development of inflammation [46, 47]. Under oxidative stress, the concentration of GSH plays an important role in the integrity and maintenance of cell structure and function. Nevertheless, GST enzymes can catalyze the binding of GSH and various oxidants during biotransformation metabolism, and so doing, protect the body against injury due to oxidative stress. Therefore, *GST* gene polymorphisms have an important role in the process of antioxidation in the lungs.

In the correlation analysis involving serum biochemical indices and TAC, the differences between the related indices across the COPD and control groups were determined first (Table 5). Significantly different ALB values, which suggested that serum albumin content in the COPD patients had decreased, were selected from this analysis. A novel index, “AC/ALB,” used to describe the antioxidant capacity per unit of albumin, was established considering the decrease of serum TAC in the COPD patients (Table 3). The new index performs a comprehensive analysis of serum antioxidant capacity and ALB content. Differences between groups (Table 6) indicate that the ratio of *Z* (M)/ALB in COPD is significantly higher than that found in the healthy group (*P* < 0.001). This demonstrates that the antioxidant capacity of per unit of albumin in the COPD patients is lower than that observed in the control individuals which suggests that the antioxidant capacity of protein has changed in COPD patients. Finally, the relationship between COPD and *GSTM*1, *GSTT*1, and *Z* (M)/ALB was determined by logistic regression, thereby establishing the COPD and *Z* (M)/ALB regression model (Table 7). From the final regression parameters, it can be seen that *Z* (M)/ALB is a risk factor for COPD, with the antioxidant capacity per unit of albumin being lower in this group. Thus, it can be concluded that the antioxidant capacity of the body is not only related to the albumin content but also closely related to the antioxidant capacity per unit of albumin. In the COPD patients, albumin content and the antioxidant activity per unit of albumin decreases and eventually has a negative effect on the total antioxidant activity. In the establishment of the regression model, *GSTM*1 and *GSTT*1 gene polymorphisms factors were excluded. This suggests that *Z* (M)/ALB is the most important factor in the occurrence of COPD when compared to the presence of the *GST* gene polymorphisms.

In summary, antioxidant capacity plays an important role in the occurrence and development of COPD. Owing to their antioxidation properties, GST enzymes are closely related to the pathogenesis of COPD. However, the deletion of the *GSTM*1 and *GSTT*1 genes may increase the oxidative stress damage to the body. This affects the catalytic function of the corresponding proteases and impacts on the antioxidant capacity of the lungs. Similarly, the antioxidant capacity per unit of albumin plays a decisive role in the TAC. Therefore, the two *GST* gene polymorphisms and the antioxidant capacity per unit of albumin jointly determine the TAC of the individual, which ultimately influences the incidence of COPD.

Including the Dalian Han people as the research population, this study performed case-control research to (1) detect the expression of antioxidase encoding genes (*GSTM*1 and *GSTT*1), (2) explore the relationship between gene deletion individuals and their susceptibility to COPD, and (3) investigate the relationship between antioxidant capacity, genes, and the presence of COPD. The principal components influencing serum TAC were analyzed by considering the clinical test indices, and the function of *GST*s genetic variation and serum albumin on the pathogenesis of COPD was explored. In view of the molecular mechanism, these results provide evidence for a new genetic marker for COPD mechanism research and clinical treatment.

## Figures and Tables

**Table 1:** The clinical characteristics and spirometric parameters of COPD patients.

Symptoms or parameters	Values
Cough	100 (%)
Expectoration	100 (%)
Dyspnea	79 (%)

FEV1/FVC	0.60 ± 0.06 (mean ± SD)
FEV1% estimated value	69.56 ± 7.91 (mean ± SD)

**Table 2:** Comparison of *GSTM*1 and *GSTT*1 gene polymorphism distributions in the COPD and control groups.

*GST*	COPD group	Control group	*χ* ^2^	*P*
*GSTM*1 (+)	13 (39.4%)	21 (63.6%)	3.882	0.049
*GSTM*1 (−)	20 (60.6%)	12 (36.4%)		

*GSTT*1 (+)	12 (36.4%)	21 (63.6%)	4.909	0.027
*GSTT*1 (−)	21 (63.6%)	12 (36.4%)		

**Table 3:** Comparison of *Z* values detected using three methods in the COPD and control groups.

*Z* value	COPD group	Control group	*t*	*P*
*Z* (I_2_/KI)	1.021 ± 1.207	0.000 ± 1.000	3.741	<0.001
*Z* (KMnO_4_)	1.622 ± 1.217	−0.000 ± 1.000	5.914	<0.001
*Z* (H_2_O_2_)	1.220 ± 1.075	0.000 ± 1.000	4.771	<0.001

*Z* (M)	1.293 ± 1.000	0.018 ± 0.812	5.686	<0.001

**Table 4:** Relationship of TAC and *GST*s gene polymorphisms.

*GST*	*N*	*Z* (M)		*t*	*Ρ*
		$\bar{\chi }$	SD		
*GSTM*1 (+)	34	0.178	0.988	4.000	<0.001
*GSTM*1 (−)	32	1.163	1.012		

*GSTT*1 (+)	33	0.112	0.948	4.545	<0.001
*GSTT*1 (−)	33	1.200	0.996		

**Table 5:** *t*-test of serum-related indices in the COPD and control groups.

Indices	COPD group	Control group	*t*	*P*
GLU	5.882 ± 1.945	5.430 ± 0.711	1.254	0.214
ALB	37.794 ± 5.763	45.294 ± 2.290	−6.947	<0.001
TP	66.479 ± 13.669	73.964 ± 3.511	−3.047	0.003

GLU: glucose; ALB: albumin; TP: total protein.

**Table 6:** Comparison of *Z* (M)/ALB in the COPD and control groups.

Group	$\bar{\chi }$	SD	*t*	*P*
COPD	0.036	0.028	6.128	<0.001
Control	0.000	0.174		

**Table 7:** Parameter estimation and test results of logistic regression.

		*B*	S.E.	Wald	df	Sig.	Exp (*B*)
Step 3	Z (M)/ALB	69.816	17.740	15.489	1	<0.001	2.093E30
	Constant	−1.104	0.401	7.570	1	0.006	0.332

*GSTM1* and *GSTT1* gene polymorphisms factors had been excluded from the regression equation.
